# Cascaded *p–d* Orbital Hybridization Interaction in Ultrathin High‐Entropy Alloy Nanowires Boosts Complete Non‐CO Pathway of Methanol Oxidation Reaction

**DOI:** 10.1002/advs.202309813

**Published:** 2024-03-14

**Authors:** Yipin Lv, Pei Liu, Ruixin Xue, Qiudi Guo, Jinyu Ye, Daowei Gao, Guangce Jiang, Shiju Zhao, Lixia Xie, Yunlai Ren, Pengfang Zhang, Yao Wang, Yuchen Qin

**Affiliations:** ^1^ College of sciences Henan Agricultural University Zhengzhou Henan 450000 P. R. China; ^2^ School of Chemistry and Chemical Engineering University of Jinan Jinan 250022 P. R. China; ^3^ College of Chemistry and Chemical Engineering Xiamen University Xiamen Fujian 361005 P. R. China; ^4^ Shandong Provincial Key Laboratory of Chemical Energy Storage and Novel Cell Technology Liaocheng University Liaocheng 252000 P. R. China; ^5^ Key Laboratory of Synthetic and Biological Colloids Ministry of Education School of Chemical and Material Engineering International Joint Research Center for Photoresponsive Molecules and Materials Jiangnan University Wuxi 214122 P. R. China

**Keywords:** cascaded *p–d* orbital hybridization, high‐entropy alloy, methanol oxidation reaction, non‐CO pathway, ultrafine nanowires

## Abstract

Designing high efficiency platinum (Pt)‐based catalysts for methanol oxidation reaction (MOR) with high “non‐CO” pathway selectivity is strongly desired and remains a grand challenge. Herein, PtRuNiCoFeGaPbW HEA ultrathin nanowires (HEA‐8 UNWs) are synthesized, featuring unique cascaded *p*–*d* orbital hybridization interaction by inducing dual *p*‐block metals (Ga and Pb). In comparison with Pt/C, HEA‐8 UNWs exhibit 15.0‐ and 4.2‐times promotion of specific and mass activity for MOR. More importantly, electrochemical in situ FITR spectroscopy reveals that the production/adsorption of CO (CO^*^) intermediate is effectively avoided on HEA‐8 UNWs, leading to the complete “non‐CO” pathway for MOR. Theoretical calculations demonstrate the optimized electronic structure of HEA‐8 UNWs can facilitates a lower energy barrier for the “non‐CO” pathway in the MOR.

## Graphical abstract

The PtRuNiCoFeGaPbW high‐entropy alloyed ultrathin nanowires (HEA‐8 UNWs) feature unique cascaded *p*–*d* orbital hybridization interaction by inducing dual *p*‐block metals (Ga and Pb). The optimized electronic structure of HEA‐8 UNWs effectively suppress the formation of ^*^CO intermediates, resulting in outstanding performance of methanol electro‐oxidation through the “non‐CO” pathway.

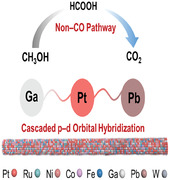

## 1 Introduction

Methanol oxidation reaction (MOR) takes place on the anode of direct methanol fuel cells (DMFCs), which could efficiently convert chemical energy into electric energy without byproducts.^[^ ^1^, ^2^, ^3^, ^4^ ^]^ Platinum (Pt) based nanostructures have been demonstrated to be one of the most efficient electrocatalysts for MOR.^[^ ^5^, ^6^, ^7^, ^8^, ^9^ ^]^ In principle, MOR follows the dual pathway mechanism including “CO” pathway and “non‐CO” pathway. In “CO” pathway of MOR, CO intermediate (CO^*^) would be produced and further oxidated to CO_2_. In contrast, formic acid (HCOOH^*^) would replace CO as the intermediate and further dehydrogenate to CO_2_ via “non‐CO” pathway.^[^ ^10^ ^]^ However, “CO” pathway is predominant at most traditional Pt based electrocatalysts and the strong Pt‐CO^*^ interaction generally leads to the easily poisoning on Pt surface. Therefore, improving the selectivity of “non‐CO” pathway on Pt‐based catalysts can effectively protect the active sites during MOR process, promoting the practical application.

High‐entropy alloys (HEAs) have attracted increasing attention in electrocatalysis owing to their unique properties.^[^ ^11^, ^12^, ^13^, ^14^, ^15^ ^]^ HEAs generally contain five or more main elements. The multi‐element composition confers extraordinary electronic structure, which could enhance the catalytic performance in various electrocatalytic reactions.^[^ ^16^, ^17^ ^]^ Despite the great performance of HEAs being achieved, most Pt‐based HEAs consist of Pt and multiple *d‐*block metals (Co, Ni, Fe, Cu, etc.), which limit the further modulation of electronic structure. Recently, unconventional *p–d* orbital hybridization interaction could be induced by alloying Pt with *p‐*block metal, affording fascinating performance toward various reactions such as oxygen reduction reaction (ORR), ethanol/ glycol electrooxidation, CO_2_ conversion, etc.^[^ ^18^, ^19^, ^20^, ^21^, ^22^ ^]^ Therefore, we anticipate that Pt‐based HEAs with a cascaded *p–d* orbital hybridization could reduce the binding strength of CO^*^ and improve the “non‐CO” pathway selectivity during MOR process.

Herein, we fabricated PtRuNiCoFeGaPbW HEA ultrathin nanowires (HEA‐8 UNWs) featuring a cascaded *p–d* orbital hybridization interaction by inducing dual *p‐*block metals (Ga and Pb). Owing to the optimized electronic structure and high‐entropy effect, HEA‐8 UNWs exhibit efficient MOR performance. The specific and mass activity of HEA‐8 UNWs are 8.56 mA cm^−2^ and 2.61 mA µg_Pt_^−1^, which are 15.0 and 4.2 times higher than that of commercial Pt/C. Importantly, the in situ electrochemical Fourier transform infrared (FTIR) analysis indicates the absence of CO^*^ peaks on HEA‐8 UNWs. It suggests that the methanol molecules can be oxidized to CO_2_ via the complete “non‐CO” pathway on HEA‐8 UNWs. Density functional theory (DFT) calculations reveal that the unique cascaded *p–d* orbital hybridization and synergistic effect of multiple elements in HEA‐8 UNWs effectively reduce the CO^*^ adsorption and strengthen OH^*^ adsorption. Additionally, the comparison of surface reaction energy barriers confirms a lower energy barrier for the “non‐CO” pathway in the MOR when utilizing the HEA‐8 UNWs catalyst. This research offers a methodical strategy for the design of efficient Pt‐based catalysis for MOR, shedding light on the atomic‐level mechanisms underlying the reaction pathway.

## 2 Results and Discussion

In a typical synthesis of HEA‐8 UNWs, platinum(II) acetylacetonate (Pt(acac)_2_), ruthenium(III) acetylacetonate (Ru(acac)_3_), nickel(II) acetylacetonate (Ni(acac)_2_), cobalt(II) acetylacetonate (Co(acac)_2_), iron(III) acetylacetonate (Fe(acac)_3_), tungsten carbonyl (W(CO)_6_), glucose, and dodecyl trimethyl ammonium chloride (DTAC) are dissolved in oleylamine under vigorous stirring and sonication consecutively. Then the resulting solution is transferred to a round‐bottom flask and heated at 220 ^о^C in oil bath for 3 h. After that, the oleylamine solution of plumbum(II) acetylacetonate (Pb(acac)_2_) and gallium(III) acetylacetonate (Ga(acac)_3_) is added slowly into aforementioned mixture at 170 ^о^C in oil bath for 3 h. Finally, HEA‐8 UNWs are obtained after centrifugation and purification. Moreover, PtRuNiCoFeW UNWs (HEA‐6 UNWs) without *p–d* orbital hybridization are also fabricated as reference by the similar method.

**Figure 1:**
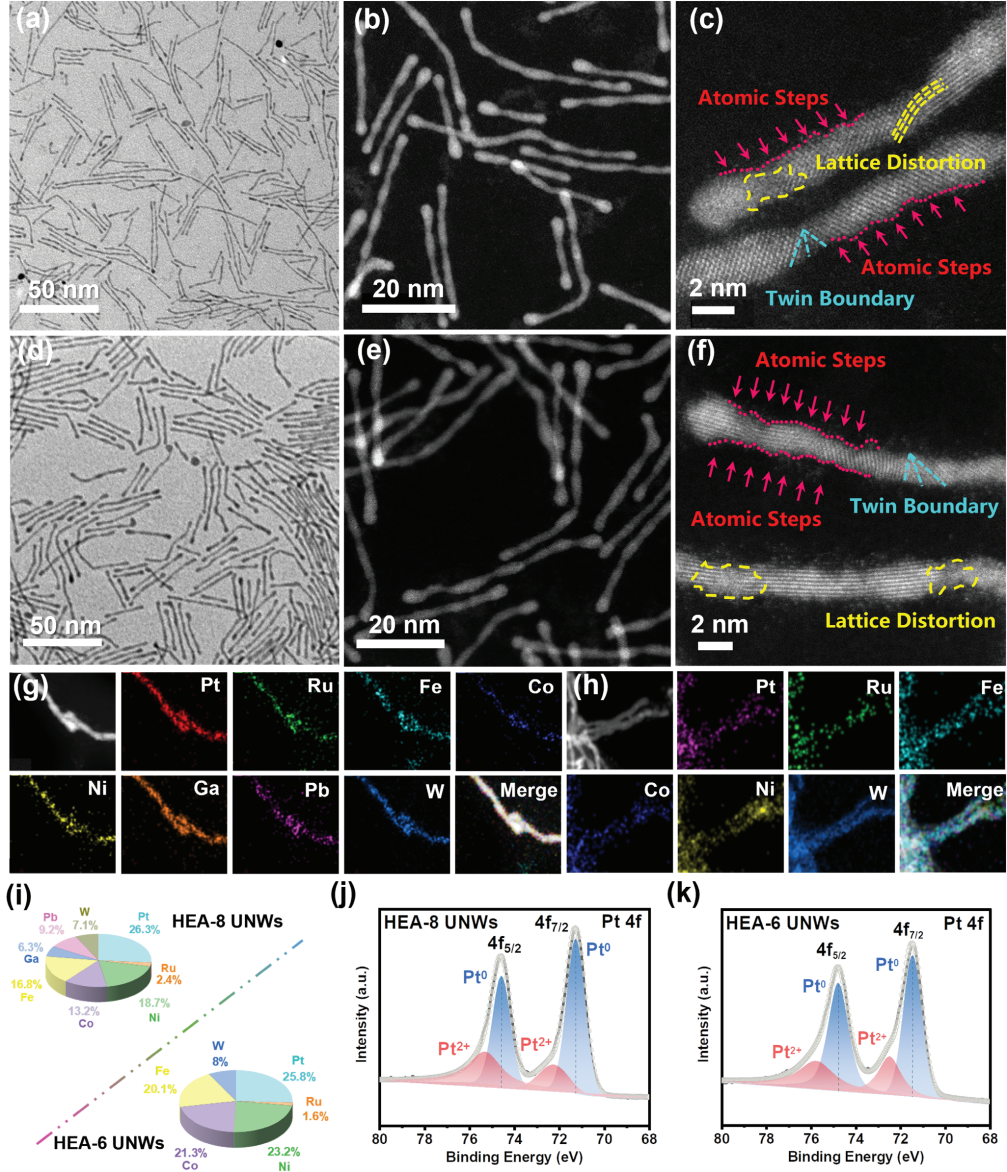
a) TEM and b) HAADF–STEM images of HEA‐8 UNWs. c) Aberration‐corrected STEM image of HEA‐8 UNWs. d) TEM and e) HAADF–STEM images of HEA‐6 UNWs. f) Aberration‐corrected STEM image of HEA‐6 UNWs. HAADF–STEM and EDS mapping images of g) HEA‐8 UNWs and h) HEA‐6 UNWs. i) The components of HEA‐8 UNWs and HEA‐6 UNWs. The Pt 4f XPS spectra of j) HEA‐8 UNWs and k) HEA‐6 UNWs.

The representative transmission electron microscopy (TEM) and high‐angle annular dark‐field scanning TEM (HAADF–STEM) images reveal the ultrafine 1D structure of HEA‐8 UNWs (**Figure** **1**a,b). The average length of HEA‐8 UNWs is ≈50 nm and the width is ≈2 nm, approximately eight atomic layers wide (Figure S1, Supporting Information). Aberration‐corrected STEM was employed to further characterize the ultrathin structure in detail. As shown in Figure 1c, abundant lattice distortion, twin boundaries, and atomic steps exist on HEA‐8 UNWs. These defects are highly desirable for enhancing MOR performance.^[^ ^23^ ^]^ The energy‐dispersive X‐ray spectroscopy (EDS) elemental mapping images display the uniform distribution of these eight metallic elements and Pt/Ru/Ni/Co/Fe/Ga/Pb/W = 25.5/1.5/19.9/12.5/18.0/ 5.8/10.4/6.4 (Figure 1g), which is close to the result of 26.3/2.4/18.7/13.2/16.8/6.3/9.2/7.1 (Figure 1i) measured by optical emission spectroscopy (ICP–OES). To compared with HEA‐8 UNWs, HEA‐6 UNWs were prepared without the dual *p*‐block metals (Pb and Ga). Figure 1d,e shows HEA‐6 UNWs exhibiting a similar ultrafine nanowire structure. The width of HEA‐6 UNWs is closed to that of HEA‐8 UNWs, but the length of HEA‐6 UNWs (≈38 nm, Figure S2, Supporting Information) is shorter than that of HEA‐8 UNWs (≈50 nm). HEA‐6 UNWs also have the lattice distortion with plentiful atomic steps and twin boundaries (Figure 1f). Meanwhile, Pt, Ru, Ni, Co, Fe, and W are distributed uniformly on HEA‐6 UNWs confirmed by EDS elemental mapping. The ratio of elements detected by EDS elemental mapping (Pt/Ru/Ni/Co/Fe/W = 25.5/1.4/25.7/20.2/19.6/7.6) is consistent with the result of ICP–OES (Pt/Ru/Ni/Co/Fe/W = 25.8/1.6/23.2/21.3/20.1/8.0, Figure 1i).

The X‐ray diffraction (XRD) patterns of HEA‐8 UNWs and HEA‐6 UNWs both display the high‐crystalline face‐centered cubic (fcc) phase of Pt and the peaks shift positively relative to that of pure Pt, demonstrating the well‐alloyed structure (Figure S3, Supporting Information). The broadening of peaks might be caused by the Bragg scattering of X‐rays on rough crystal plane with abundant lattice distortion, corresponding to the results of TEM. The X‐ray photoelectron spectroscopy (XPS) reveals that both HEA‐8 UNWs and HEA‐6 UNWs exhibit the dominant metallic state (Pt^0^) of Pt. Compared to the Pt 4f_7/2_ peak of HEA‐6 UNWs at 70.47 eV, the band energy of HEA‐8 UNWs (70.21 eV) shifted negatively by 0.26 eV, demonstrating that more electrons might transfer from other elements to Pt in HEA‐8 UNWs (Figure 1j,k). Furthermore, surface valence band photoemission spectra show that an obvious downshift of *d‐*band center at HEA‐8 UNWs (−4.24 eV) could be observed in comparison with HEA‐6 UNWs (−3.31 eV), indicating the weaker adsorption for intermediates such as CO^*^ in MOR on HEA‐8 UNWs (Figure S4, Supporting Information).

**Figure 2:**
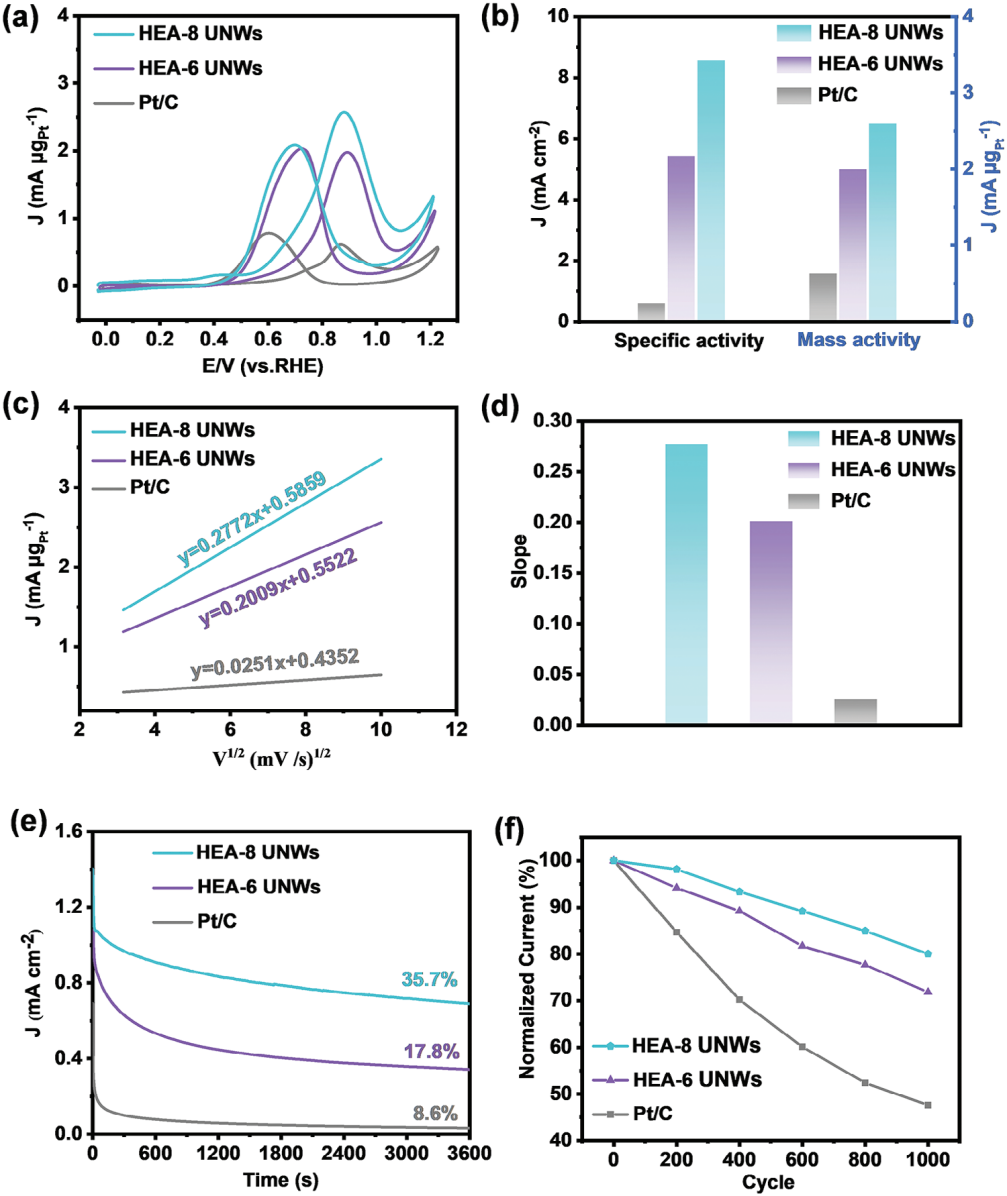
a) Cyclic voltammogram curves of HEA‐8 UNWs, HEA‐6 UNWs, and Pt/C in 0.5 m H_2_SO_4_ + 2 m CH_3_OH. b) The comparison of catalytic performances. c) The plots of forward peak current J (mA µg_Pt_^−1^) versus the square root of the scan rate (*v* ^1/2^) for MOR. d) The comparison of the slope of (c). e) *i*–*t* curves (at 0.6 V vs RHE) and f) the normalized current after various CV cycles for HEA‐8 UNWs, HEA‐6 UNWs, and Pt/C.

As a proof‐of‐concept application, the catalytic performances of HEA‐8 UNWs toward MOR were conducted. HEA‐6 UNWs and commercial Pt/C were selected as the reference catalysts. The electrochemical surface areas (ECSAs) were evaluated by cyclic voltammetry (CV) measurements in 0.5 m H_2_SO_4_ (Figure S5, Supporting Information). HEA‐8 UNWs (41.2 m^2^ g^−1^) exhibit the larger ECSA than that of HEA‐6 UNWs (26.4 m^2^ g^−1^), but lower than Pt/C (53.6 m^2^ g^−1^). **Figures** **2**a and S6 (Supporting Information) show the CVs of MOR in N_2_‐saturated acid media. The currents were normalized to ECSA and mass loading of Pt to calculate the specific activity and mass activity. Compared with HEA‐6 UNWs and Pt/C, HEA‐8 UNWs delivered the highest catalytic activity. The specific activity (mass activity) of HEA‐8 UNWs is 8.56 mA cm^−2^ (2.61 mA µg_Pt_^−1^), which is 15.0 (4.2) times higher than that of Pt/C (Figure 2b). In previous reports, PtRu was the widely used catalyst for MOR. In comparison with PtRu and other state‐of‐art catalysts, HEA‐8 UNWs also exhibit an excellent performance of MOR (Figure S7, Supporting Information).^[^ ^24^, ^25^, ^26^, ^27^, ^28^, ^29^, ^30^ ^]^ To further evaluate the catalytic properties of these catalysts, the CVs was measured at different scan rates from 10 to 100 mV s^−1^. Figure 2c shows the plots of the forward current density (*J*_m_) versus the square root of the scan rate (*v* ^1/2^). It could be observed that the square root of the scan rate (*v* ^1/2^) is linear relationship with the current density (*J*_m_). As shown in Figure 2d, HEA‐8 UNWs display the largest slope value among these catalysts, demonstrating the great enhancement of MOR kinetics on HEA‐8 UNWs. The superior MOR performance could be attributed to the optimized electronic structure and abundant defects on HEA‐8 UNWs.^[^ ^31^ ^]^ The relevant electrochemical data has been summarized in Table S1 (Supporting Information). HEA‐8 UNWs exhibited the the highest catalytic activity and low energy barrier. Moreover, the stability of catalysts was evaluated by various methods. Figure 2e displays the current of HEA‐8 UNWs could remain 35.7% of the initial value (current at 1 s) after 3600 s durability measurements, which is much higher than that of Pt/C (8.6%) and HEA‐6 UNWs (17.8%). In addition, the enhanced stability of HEA‐8 UNWs can also be demonstrated by CVs experiments. As shown in Figure 2f, HEA‐8 UNWs show the slowest decline in activity after different CVs cycles. After 1000 CVs cycles, the activity can remain 80% of the initial activity, which is obviously higher than that of Pt/C (47.6%) and HEA‐6 UNWs (71.8%). Furthermore, chronoamperometric measurements were carried out at various potentials. It was observed that HEA‐8 UNWs also displayed satisfactory durability at different potentials (Figure S8, Supporting Information). After conducting the stability test, the nanowire structure was largely preserved on HEA‐8 UNWs, but a small part is fractured. (Figure S9, Supporting Information). Table S2 (Supporting Information) showed the atomic ratio of HEA‐8 UNWs slightly changed after stability measurement. Generally, the excellent durability of Pt‐based catalysts towards MOR could be attributed to the strong CO tolerance ability. Thus, CO‐stripping voltammograms and has been employed to evaluate the CO oxidation removal ability. Figure S10 (Supporting Information) shows that the potential of CO_ad_ oxidation removal on HEA‐8 UNWs is the lowest among these three catalysts. It negatively shifts ≈70 mV compared to that of Pt/C, indicating HEA‐8 UNWs possess an excellent CO oxidation removal capacity. Besides the ability of CO oxidation removal, CO adsorption strength is another crucial factor influencing the CO tolerance capability.

**Figure 3:**
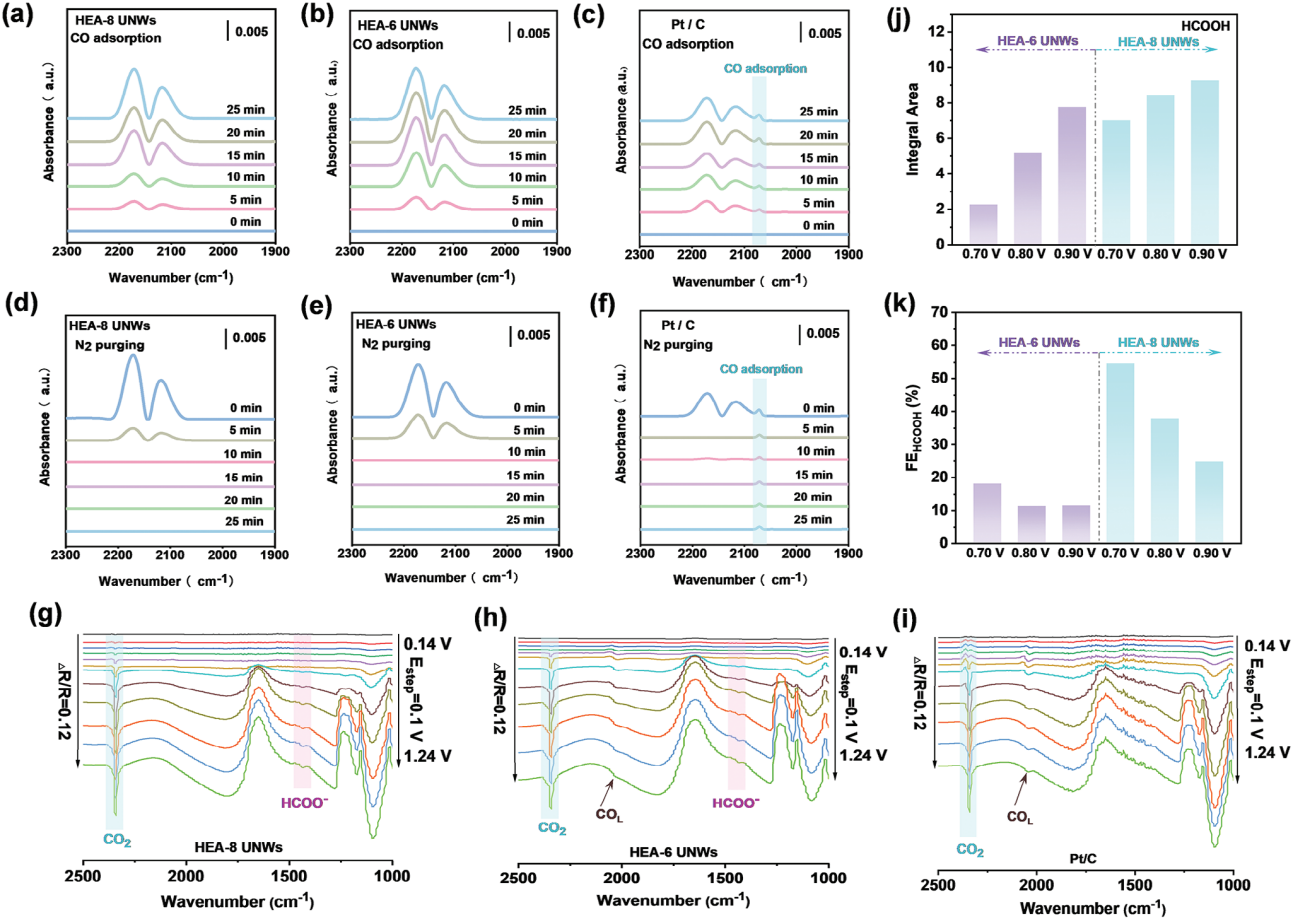
CO–DRIFTS spectra of a) HEA‐8 UNWs, b) HEA‐6 UNWs and c) Pt/C. N_2_‐purging CO–DRIFTS spectra of d) HEA‐8 UNWs, e) HEA‐6 UNWs and f) Pt/C. Electrochemical in situ FTIR spectra of g) HEA‐8 UNWs, h) HEA‐6 UNWs, and i) Pt/C. Potential dependent j) amount of HCOOH and k) Faraday efficiency (FE) on HEA‐8 UNWs and HEA‐6 UNWs.

To reveal CO adsorption, CO diffuse reflectance infrared Fourier transform spectroscopy (CO‐DRIFTS) has also been conducted. **Figure** **3**a–c shows that only CO gas peaks at ≈2175 and 2115 cm^−1^ occurred on HEA‐8 UNWs and HEA‐6 UNWs. While, the CO adsorption peak of 2074 cm^−1^ exists on Pt/C, which cannot be completely removed after N_2_ purging (Figure 3d–f), indicating the HEA‐8 UNWs and HEA‐6 UNWs possess the weaker CO adsorption strength than that of Pt/C.

For “non‐CO” pathway, ^*^OH as a crucial intermediate, is formed from water splitting in acid solution. It can interact with ^*^CHO to produce ^*^HCOOH and further dehydrogenize to CO_2_ (^*^OH+^*^CHO→^*^HCOOH→CO_2_), releasing six electrons and avoiding CO poisoning.^[^ ^32^ ^]^ While, ^*^CO would be produced in “CO” pathway by dehydrogenation of ^*^CHO and further oxide to CO_2_. To gain insights into the reaction pathway of MOR on HEA‐8 UNWs, HEA‐6 UNWs, and Pt/C at the molecular‐level, electrochemical in situ FTIR measurements were carried out.

Figure 3g–i all display the obvious asymmetric stretch vibration of CO_2_ at ≈2345 cm^−1^, demonstrating CO_2_ is the dominated product of MOR on these various electrocatalysts. The absorption peak of linearly bonded CO (CO_L_) at ≈2035 cm^−1^ is observed on HEA‐6 UNWs and Pt/C, revealing “CO” pathway occurred on both HEA‐6 UNWs and Pt/C during MOR process. Besides the peak of CO_L_, it is noted that the vibration peak of COO^−^ at ≈1430 cm^−1^ can be detected on HEA‐6 UNWs from 0.74 V (RHE), meaning HCOOH is another reactive intermediate on HEA‐6 UNWs at higher potential. This result suggests that methanol molecules can be oxidized to CO_2_ on HEA‐6 UNWs via dual pathway at various potential. Specifically, “CO” pathway is dominant when the potential is below 0.74 V. As the potential increased (> 0.74 V), “non‐CO” pathway will accompany “CO” pathway on HEA‐6 UNWs during MOR process. However, for the HEA‐8 UNWs, no CO peaks can be detected and only the peak of COO^−^ can be observed in operando FTIR spectrogram, confirming “non‐CO” pathway is predominant on HEA‐8 UNWs in MOR process.

Moreover, the amount of HCOOH formed on HEA‐6 UNWs and HEA‐8 UNWs during MOR was detected to further reveal the enhancement of “non‐CO” pathway selectivity. The products after chronoamperometric measurements for 2 h at various potentials were collected and further analyzed by high performance liquid chromatography (HPLC). As shown in Figure S11 (Supporting Information) and Figure 3j, a distinct difference of HCOOH yield can be observed over HEA‐6 UNWs at different potential. Only a small amount of HCOOH can be obtained at 0.70 V (RHE), demonstrating “CO” pathway is predominant on HEA‐6 UNWs at lower potential. As the potential increases, more HCOOH can be generated and participated in the “non‐CO” pathway over HEA‐6 UNWs. Quite different from HEA‐6 UNWs, high HCOOH yield can be detected even at low potential over HEA‐8 UNWs, illustrating “non‐CO” pathway is predominant on HEA‐8 UNWs. Furthermore, the Faraday efficiency of HCOOH (FE_HCOOH_) on HEA‐8 UNWs is much higher than that of HEA‐6 UNWs (Figure 3k), especially at lower potential. It confirms that HEA‐8 UNWs possess the higher “non‐CO” pathway selectivity during MOR. It is worth noting that the FE_HCOOH_ of HEA‐8 UNWs reduces obviously with the uplifted potentials, suggesting that more HCOOH, as the reactive intermediates, were further oxidized to CO_2_ at higher potential_._^33^ Thus, the HCOOH electrooxidation reaction (FOR) was also investigated on HEA‐8 UNWs. As shown in Figure S12 (Supporting Information), HEA‐8 UNWs exhibit the superior FOR performance, demonstrating the strong ability of formic acid oxidation.

**Figure 4:**
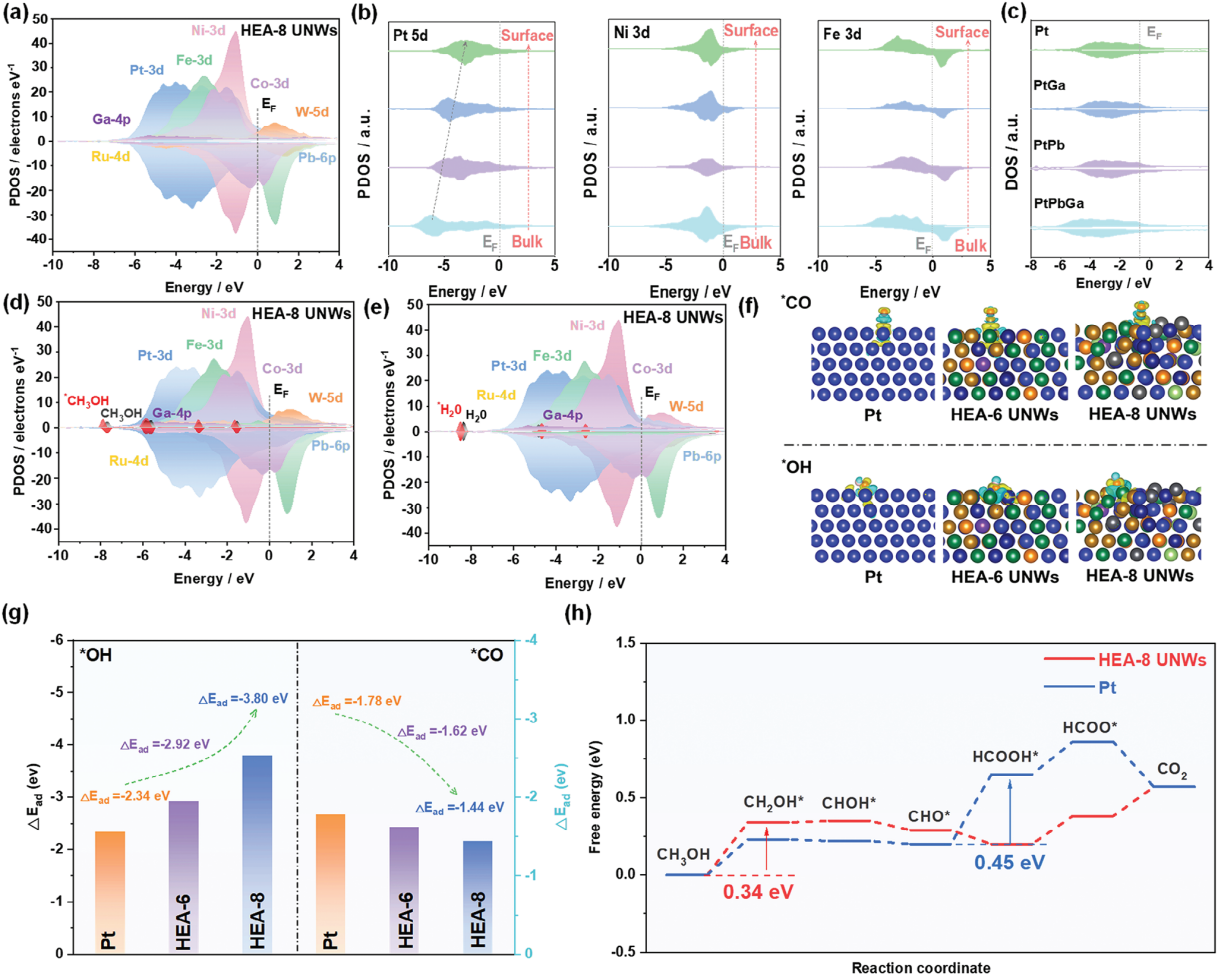
The PDOSs of a) HEA‐8 UNWs. b) The site‐dependent PDOSs of Pt Co and Fe. c) The DOS of Pt, PtGa, PtPb, and PtGaPb in HEA‐8 UNWs. The PDOS of d) CH_3_OH adsorption and e) H_2_O adsorption. f) The charge density difference of CO adsorption and OH adsorption. g) The adsorption energy of ^*^OH and ^*^CO on three different catalysts. h) The variation of Gibbs free energy of the MOR.

DFT calculations were further conducted to shed light on the regulation mechanism of MOR pathway on HEA‐8 UNWs. HEA‐8 UNWs and HEA‐6 UNWs models were constructed based on the ICP results. Pt (111) surface was also modelled as reference (Figure S13–S15, Supporting Information). At first, the partial projected density of states (PDOSs) was calculated to reveal the detailed electronic structure of HEA‐8 UNWs. As shown in **Figure** **4**a, we notice the obvious overlaps between orbitals, demonstrating the strong interaction between various elementals in HEA UNWs. In particular, the Ga‐4*p* and Pb‐6*p* band matches well with the *d‐*orbitals of multiple metals, indicating the cascaded *p‐d* orbital hybridization interaction in HEA‐8 UNWs. The sharp Ni‐3*d* orbital peak at −1.07 eV is evident, which is conducive to promote the ^*^OH adsorption. The Co‐3*d* orbital is closed to the Ni‐3d orbital. Thus, both Co and Ni can be employed as the electron depletion centers to assist stabilize the adsorption intermediates of MOR. It should be noted that Pt‐5*d* orbitals occupies the deepest position to E_F_ (≈4.41 eV), indicating Pt is employed as the electron reservoir for electrocatalytic process.^[^ ^34^ ^]^ Ru‐4*d* and W‐5*d* orbitals display a broad band and cross the Fermi level, further accelerating the electron transfer from HEA‐8 UNWs surface to adsorbed intermediates. In addition, the average number of transferred electrons was calculated based on the Bader charge analysis (Table S3, Supporting Information). The Pt and Ru atoms obtain electrons and other metals lose electrons confirming the strong interaction between different metals. The strong electron correlation will facilitate electron transfer in HEA‐8 UNWs and optimize the electronic structure of active sites. Moreover, a detailed investigation on site‐dependent electronic structure of elements in HEA‐8 UNWs was also conducted. Figure 4b displays an evident upshift to the Fermi level of Pt‐5*d* center from bulk to surface, indicating the electron transfer and enhanced intermediates adsorption on the surface. Meanwhile, the Ni‐3*d* and Fe‐3*d* centers has not changed much from bulk to surface, suggesting the highly localized electron density on surface of HEA‐8 UNWs. It can facilitate the charge migration and promote the interaction with ^*^OH.^[^ ^35^ ^]^ Furthermore, we interpret the cascaded *p–d* orbital hybridization of Pt‐5*d* with Ga‐4*p* and Pb‐6*p* in HEA‐8 UNWs. As shown in Figure 4c, the cascaded *p–d* orbital hybridization enriches the electron density near the Fermi level, contributing to the enhanced site‐to‐site electron transfer among active sites in HEA‐8 UNWs.^[^ ^36^ ^]^

This optimized electronic structure of HEA‐8 UNWs is conducive to deliver a superior MOR performance and inhibit the production of poisonous CO intermediate. For MOR process, the initial CH_3_OH adsorption is a vital factor for the MOR performance. As shown in Figure 4d, the *s, p* orbitals of ^*^CH_3_OH shift negatively in comparison with free CH_3_OH molecule. It confirms the active electron transfer from HEA‐8 UNWs surface to CH_3_OH, resulting in the stable adsorption for the efficient MOR process. Moreover, considering ^*^OH is the crucial intermediate for “non‐CO” pathway, which is achieved by water splitting. The adsorption of water on HEA‐8 UNWs was also investigated. Similarly, a distinct downshift of *s, p* orbitals in ^*^H_2_O is observed, indicating the strong interaction between H_2_O and HEA‐8 UNWs surface (Figure 4e). This is beneficial for the electron and proton transfer and boost the water dissociation process.^[^ ^34^ ^]^

Figure 4f shows the electronic redistribution of ^*^CO and ^*^OH adsorption on Pt/C, HEA‐6 UNWs, and HEA‐8 UNWs. The electron transfer on HEA‐6 UNWs and HEA‐8 UNWs is more pronounced than Pt/C. Meanwhile, the adsorption energy of ^*^CO and ^*^OH on various catalysts were calculated. As shown in Figure 4g, HEA‐8 UNWs exhibit the strongest ^*^OH adsorption and the weakest ^*^CO adsorption among these catalysts. The adsorption energy of ^*^OH (−3.80 eV) is much larger than that of ^*^CO (−1.44 eV) on HEA‐8 UNWs, which can be attributed to the optimized electronic structure modified by cascaded *p–d* orbital hybridization and synergistic effect of multiple metals on HEA‐8 UNWs. This result indicates ^*^OH can be preferentially and stably adsorbed on HEA‐8 UNWs, which is in favor of “non‐CO” pathway rather than “CO” pathway of MOR.^[^ ^31^ ^]^ Then, the variation of Gibbs free energy for “non‐CO” pathway on HEA‐8 UNWs and Pt/C were further calculated (Figure 4h). We notice that HEA‐8 UNWs possess a smoother “non‐CO” pathway in comparison with Pt/C. The largest energy cost is only 0.34 eV for HEA‐8 UNWs, which is obviously lower than that of Pt/C (0.45 eV). These calculated results clearly demonstrate the higher possibility for “non‐CO” pathway on HEA‐8 UNWs during MOR process, in good agreement with in situ electrochemical FTIR measurements.

## 3 Conclusion

In summary, HEA‐8 UNWs have been successfully prepared though a facile wet–chemical approach, featuring the ultrafine 1D structure with abundant low‐coordination atoms. Importantly, HEA‐8 UNWs possess a unique cascaded *p–d* orbital hybridization interaction and synergistic effect of multiple metals. These properties effectively inhibit the production of ^*^CO intermediates and deliver an excellent MOR performance via “non‐CO” pathway. The specific activity (mass activity) of HEA‐8 UNWs is 15.0 (4.2) times higher than that of Pt/C. Especially, electrochemical in situ FTIR demonstrates that no peaks of ^*^CO intermediates can be detected on HEA‐8 UNWs during MOR process, suggesting the complete “non‐CO” pathway for MOR. DFT results reveal the optimized electronic structure of HEA‐8 UNWs can decrease the energy barrier of the “non‐CO” pathway in the MOR. This work suggests that fabricating HEA catalysts with cascaded *p–d* orbital hybridization will lighten the further design of high efficiency Pt‐based electrocatalysts.

## Conflict of Interest

The authors declare no conflict of interest.

## Supporting information

Supporting Information

## Data Availability

The data that support the findings of this study are available from the corresponding author upon reasonable request.
