# Supplementary material for: Cascaded p–d Orbital Hybridization Interaction in Ultrathin High‐Entropy Alloy Nanowires Boosts Complete Non‐CO Pathway of Methanol Oxidation Reaction
Source: Adv Sci (Weinh). 2024 Mar 14;11(19):2309813. doi: 10.1002/advs.202309813 (PMC11109631; doi:10.1002/advs.202309813)
Supplement: Supplementary file 1 — Supporting Information [file ADVS-11-2309813-s001.pdf]

ADVANCED SCIENCE
Open Access

Supporting Information

for *Adv. Sci.*, DOI 10.1002/advs.202309813

Cascaded *p–d* Orbital Hybridization Interaction in Ultrathin High-Entropy Alloy Nanowires Boosts Complete Non-CO Pathway of Methanol Oxidation Reaction

Yipin Lv, Pei Liu, Ruixin Xue, Qiudi Guo, Jinyu Ye, Daowei Gao, Guangce Jiang, Shiju Zhao, Lixia Xie, Yunlai Ren, Pengfang Zhang, Yao Wang* and Yuchen Qin**

Supporting Information

Cascaded *p–d* Orbital Hybridization Interaction in Ultrathin High–entropy Alloy Nanowires Boosts Complete Non–CO Pathway of Methanol Oxidation Reaction

Yipin Lv,[1,2#] Pei Liu,[1#] Ruixin Xue,[2] Qiudi Guo,[1] Jinyu Ye,[5] Daowei Gao,[2] Guangce Jiang,[1] Shiju Zhao,[1] Lixia Xie,[1] Yunlai Ren,[1] Pengfang Zhang,[3] Yao Wang,[4*] Yuchen Qin[1*]*

1 College of sciences, Henan Agricultural University, Zhengzhou, Henan 450000 (P. R. China)

2 School of Chemistry and Chemical Engineering, University of Jinan, Jinan 250022, PR China.

3 Shandong Provincial Key Laboratory of Chemical Energy Storage and Novel Cell Technology, Liaocheng University, Liaocheng 252000, P. R. China

4 Key Laboratory of Synthetic and Biological Colloids, Ministry of Education, School of Chemical and Material Engineering, International Joint Research Center for Photoresponsive Molecules and Materials, Jiangnan University, Wuxi, 214122, China

5 college of Chemistry and Chemical Engineering, Xiamen University Xiamen, Fujian 361005 (P. R. China)

#: Yipin Lv and Pei Liu contribute equally to this work.

Experimental Procedures

Chemicals and Reagents. Platinum(II) acetylacetonate (Pt(acac)$_2$, 98%), Ruthenium(III) acetylacetonate (Ru(acac)$_3$, 98%), Iron (III) acetylacetonate (Fe(acac)$_3$, 98%), Nickle(II) acetylacetonate (Ni(acac)$_2$, 98%), Cobalt(II) acetylacetonate (Co(acac)$_2$, 98%), Plumbum(II) acetylacetonate (Pb(acac)$_2$, 98%), galliumand(II) acetylacetonate (Ga(acac)$_2$, 98%) and tungsten hexacarbonyl (W(CO)$_6$) were purchased from Macklin. Glucose, dodecyltrimethylammonium chloride (DTAC) and oleylamine were purchased from Sinopharm Chemical Reagent Co. Ltd. Commercial Pd/C was purchased from Sigma-Aldrich. Deionized water was used in experiments and all reagents were used as received without further purification.

Synthesis of HEA-6 UNWs. 10.0 mg Pt(acac)2, 5.0 mg Ru(acac)3, 4.0 mg Ni(acac)2, 7.0 mg Co(acac)3, 7.0 mg Fe(acac)3, 10.0 mg W(CO)6, 60.0 mg glucose, 60.0 mg glucose DTAC and solution were dissolved in 5 mL oleylamine. The glass bottle was then placed in an ultrasonic scrubber for 2 hours until all solids are fully dissolved and the solution is clear and transparent. Transfer the above solution to a 50 mL round-bottled flask and heat the mixture in an oil bath at 220°C for 3 hours to produce PtRuNiCoFeW NWs, then cool to room temperature. The cooled product was collected by centrifugation and washed three times with a cyclohexane/ethanol mixture.

Synthesis of HEA-8 UNWs. 2.0 mg Pb(acac)2, 4.0 mg Ga(acac)3 were dissolved in 2 mL oleylamine solution. Then, the glass bottles are placed in an ultrasonic scrubber and treated for 30 min until all solids are completely dissolved and the solution is clear. The above solution was slowly added to HEA-6 UNWs solutions obtained above and heated in an oil bath at 170°C for 3 hours, and then cooled to room temperature. The cooled product was collected by centrifugation and washed three times with a cyclohexane/ethanol mixture.

Electrochemical measurement. All electrochemical tests were performed on the CHI 760e using a standard three-electrode system at room temperature. The glassy carbon electrode (GCE) loaded with catalyst were used as the working electrodes, with a diameter of 3 mm. Catalyst ink was achieved by dispersing 1 mg catalyst in 1 mL ethanol in an ultrasonic bath. The GCE was polished and cleaned with alumina powder. To prepare the working electrode, 6 μL catalyst ink was spread on the GC electrode surface and dried in air, then 2 μL Nafion solution (0.05 wt%) was dropped on it.

CO stripping experiments. First, purged 0.5 M H_2SO_4 solution with N_2 for 30 minutes to remove the air in the solution. Second, CO was bubbled for 20 minutes. Then, the solution was purged with N_2 for 30 minutes to remove excess CO. Finally, CVs were performed at a scan rate of 50 $mV \cdot s^{-1}$ from -0.24 ~ 1.0 V (vs. SCE) to obtain the CO oxidation peak.

Characterization. The morphology of HEA UNWS was revealed by Tecnai G2 F20 S-Twin high-resolution transmission electron microscopy (HRTEM) operating at 200 kV and Aberration-corrected STEM (JEM-ARM300F) operating at 300 kV. X–ray photoelectron spectrum (XPS) analysis was characterized on a PHI 5000 Versaprobe system using monochromatic Al Kα radiation (1486.6 eV). The binding energies were calibrated by the C 1s peak at 284.6 eV. The X-ray diffraction (XRD) patterns of samples were conducted on a Bruker D8-advance X-ray powder diffractometer operated at voltage of 40 kV and current of 40 mA with CuK radiation (λ = 0.154056 nm). The Inductively coupled plasma optical emission spectrometry (ICP-OES) of samples was tested on IRIS Intrepid II XSP (ThermoFisher).

Electrochemical in situ Fourier transform infrared (FTIR) reflection spectroscopy. In situ electrochemical FTIR measurements were conducted using a Nexus 870 spectrometer (Nicolet) equipped with a liquid nitrogen-cooled MCT-A detector. The homemade electrochemical cell is consisted of a CaF_2 window and window and a thin layer (≤ 10 μm) between the GCE and CaF_2 window. Three-electrode system was

carried out to finish the measurements. FTIR measurements were recorded from 0.14-1.24 V (Vs. RHE) with an interval of 0.1 V. The resulting spectra were measured as the relative change in reflectivity and calculated as follows:

$\Delta R/R = (R(Ec)-R(E_R))/R(E_R)$

where R(Ec) and $R(E_R)$ are the single-beam spectra collected at the catalysts potential Ec and reference potential E_R, respectively.

Computational Details and Model. All simulations were performed on the basis of DFT calculations implemented in the Vienna ab initio simulation package (VASP) with Perdew-Burke-Ernzerhof (PBE) functional of exchange-correlation. The cut-off of the kinetic energy for the plane-wave basis sets was set to 450 eV. The convergence criterion of the self-consistent field and force were set to be 10^{-4} eV and 0.05 eV/Å for geometry optimization. A Gaussian smearing of 0.2 eV was applied during the geometry optimization and for the total energy computations. Based on the experimental results, the HEA structure has been cleaved from the (111) surface of Pt with five-layered thickness and the number of atoms is 125. The atomic arrangements of different elements are constructed randomly by following the same ratio as the experiments of $Pt_{33}Ru_3Fe_{21}Co_{16}Ni_{23}W_9Ga_8Pb_{12}$ for HEA-8 and $Pt_{32}Ru_2Fe_{25}Co_{27}Ni_{29}W_{10}$. A vacuum layer of 20 Å along the *z* direction was placed between the Pt surface and its mirror images to avoid the artificial interactions among them. The bottom atomic layer was fixed. The k-point sampling was obtained from the Gamma scheme with a (2 × 2 × 1) mesh. The adsorption energy (E_{ad}) is defined as $E_{ads} = E_{adsorbate/substrate} - E_{adsorbate} - E_{substrate}$, where $E_{adsorbate/substrate}$ is the total energy of the adsorption configuration on the corresponding catalyst surface, $E_{adsorbate}$ and $E_{substrate}$ represent the energy of adsorbates and the substrates, respectively.

The stepwise dehydrogenation of methanol on Pt surface was shown:

$CH_3OH^* \rightarrow CH_2OH^* + H^+ + e^-$

$CH_2OH^* \rightarrow CHOH^* + H^+ + e^-$

$CHOH^* \rightarrow CHO^* + H^+ + e^-$

$$CHO^* + H_2O \rightarrow HCOOH^* + H^+ + e^-$$

$$HCOOH^* \rightarrow HCOO^* + H^+ + e^-$$

$$HCOO^* \rightarrow CO_2^* + H^+ + e^-$$

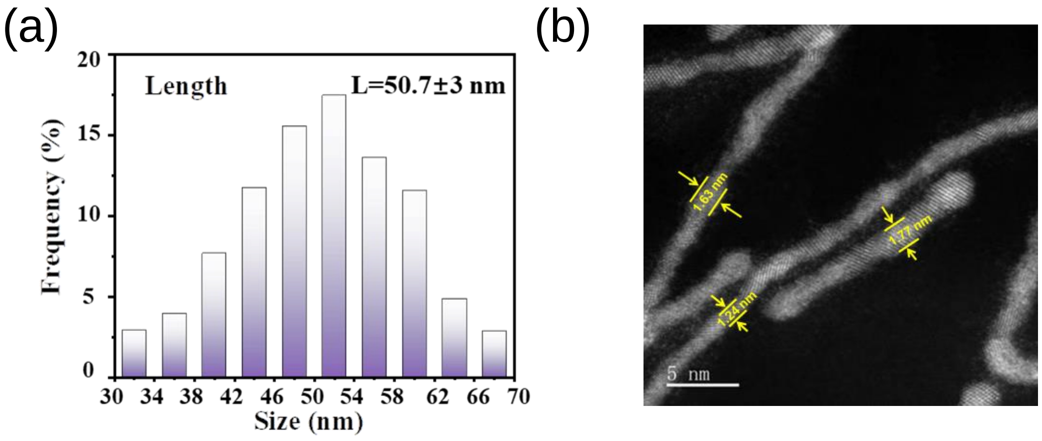

Figure S1. (a) Size-distribution histogram and (b) Aberration–corrected STEM image of HEA–8 UNWs.

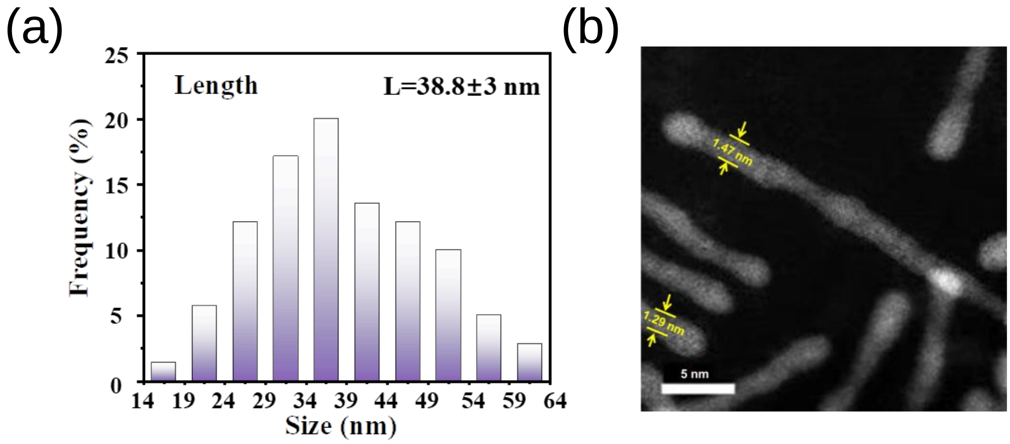

Figure S2. (a) Size-distribution histogram and (b) Aberration–corrected STEM image of HEA–6 UNWs.

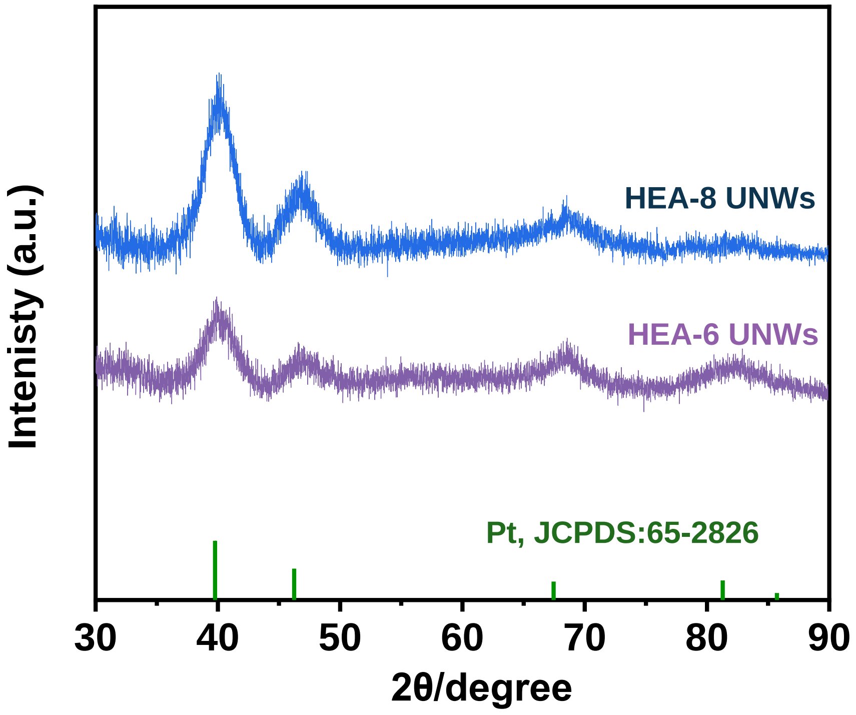

Figure S3. The XRD patterns of HEA UNWs.

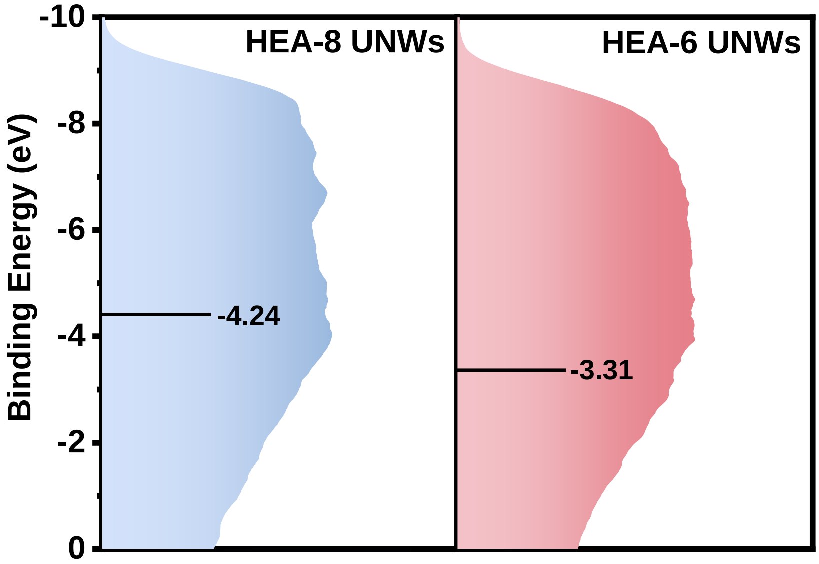

Figure S4. The d-band center of HEA UNWs.

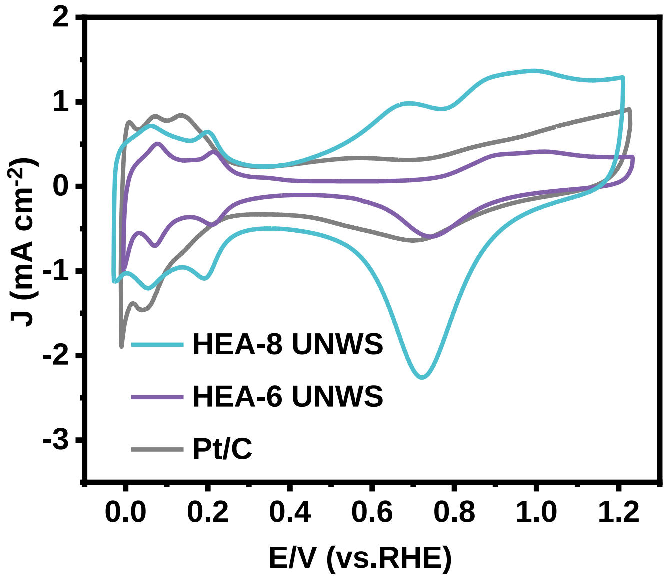

Figure S5. Cyclic voltammogram curves of HEA–8 UNWs, HEA–6 UNWs and Pt/C in 0.5 M H_2SO_4.

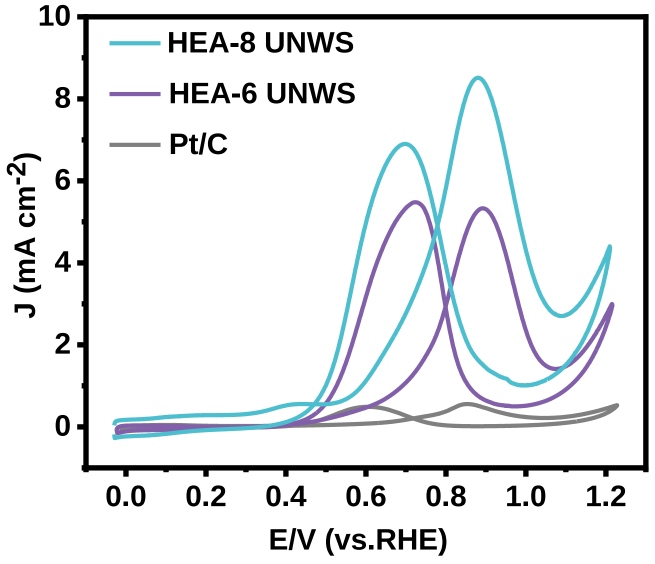

Figure S6. Cyclic voltammogram curves of HEA–8 UNWs, HEA–6 UNWs and Pt/C in 0.5 M H_2SO_4 +2 M CH_3OH.

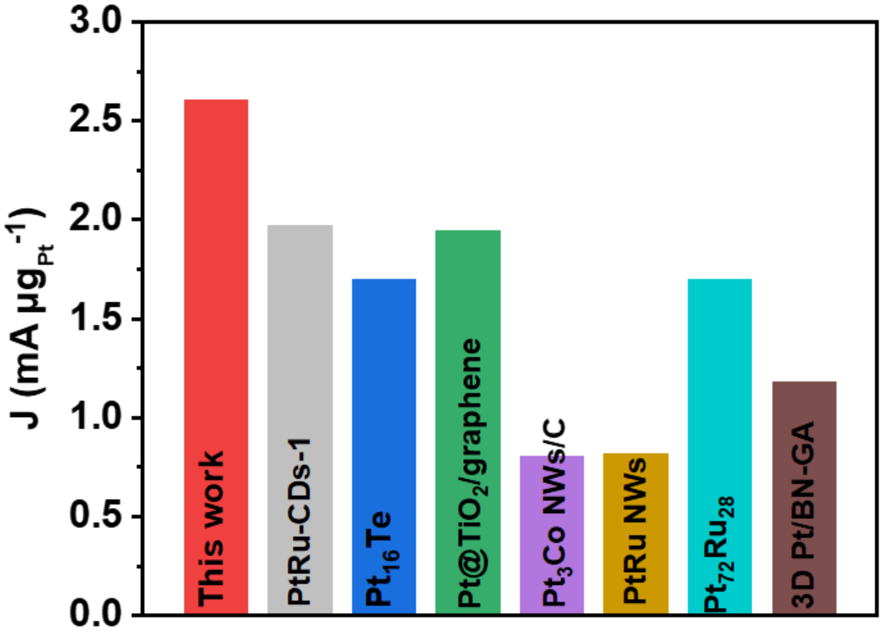

Figure S7. Comparisons of mass activity towards MOR employed in this study and some Pt-based electrocatalysts in previously reported.

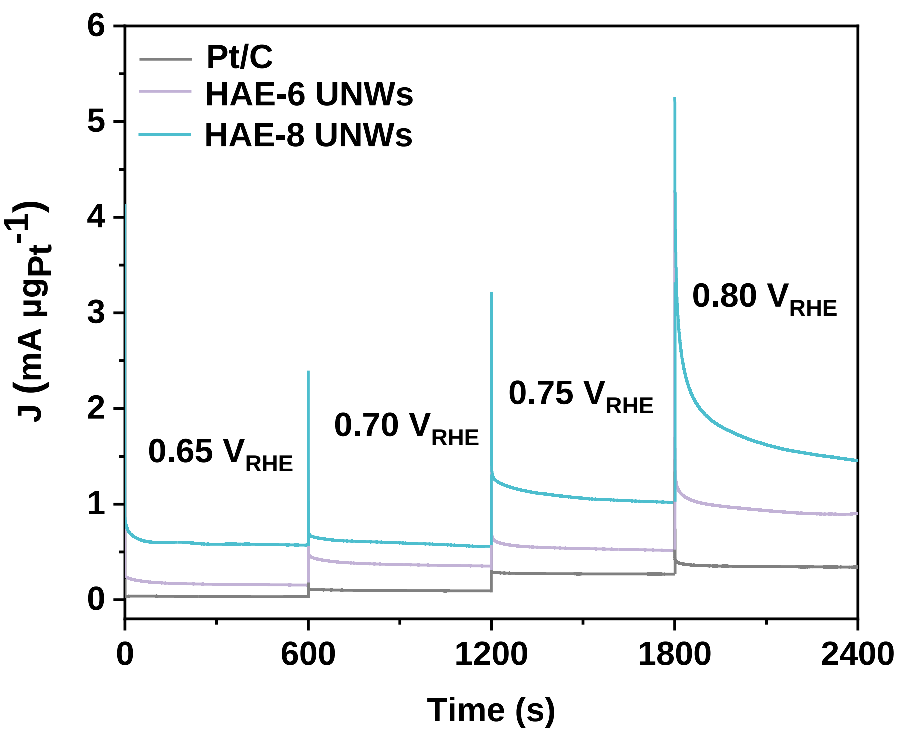

Figure S8. Chronoamperometry data at the indicated potentials in 0.5 M H_2SO_4 +2 M CH_3OH over various catalysts.

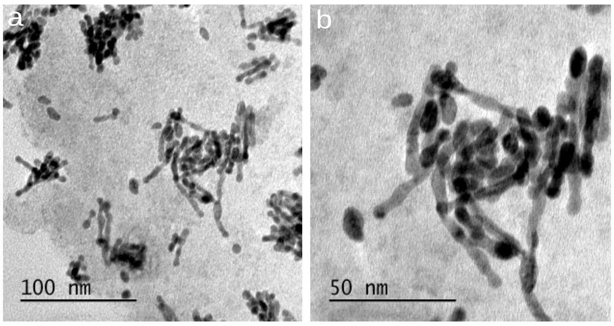

Figure S9. TEM images of HEA-8 UNWs after stability test.

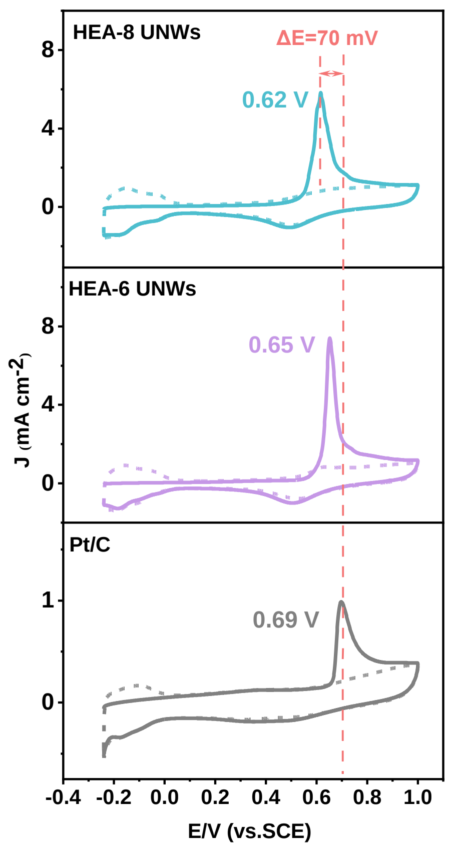

Figure S10.CO–stripping voltammograms of HEA–8 UNWs, HEA–6 UNWs and Pt/C.

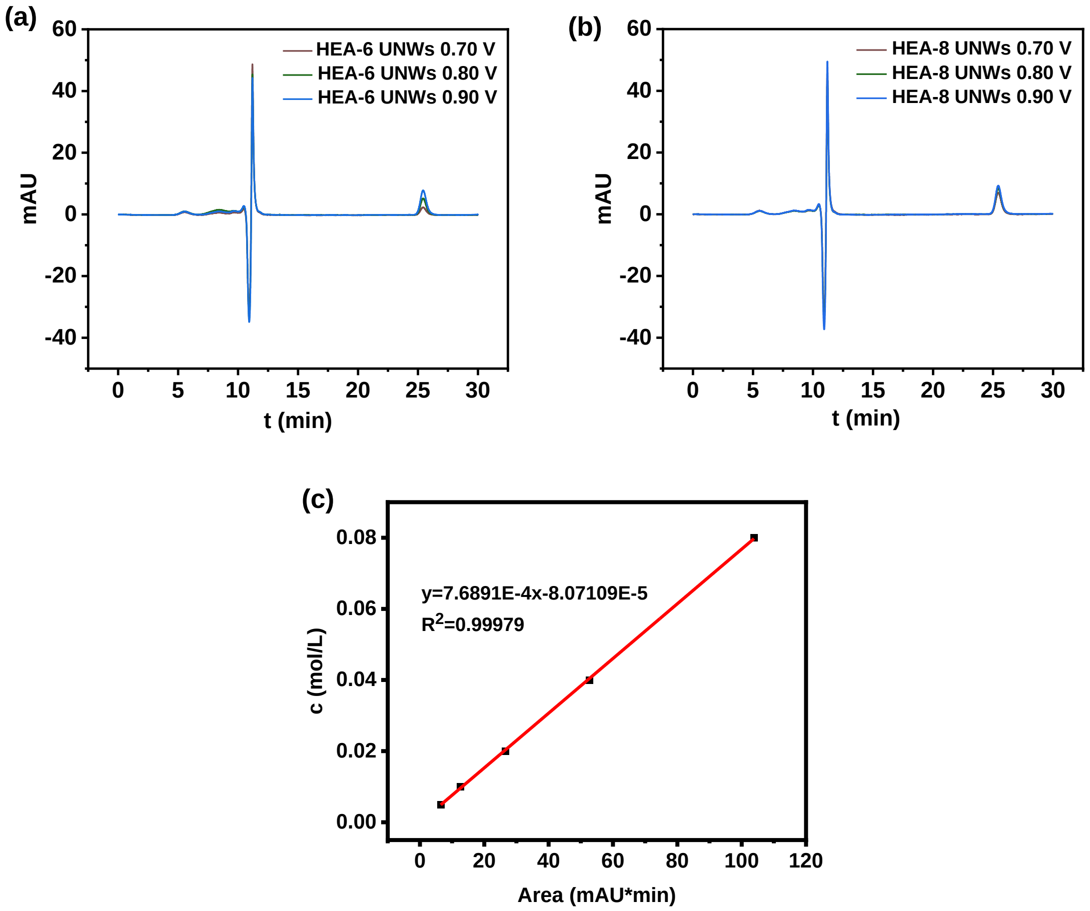

Figure S11. HPLC analysis of products after MOR on (a) HEA-6 UNWs and (b) HEA-8 UNWs at various potential. (c) The standard curves of HCOOH in HPLC for determining the concentration.

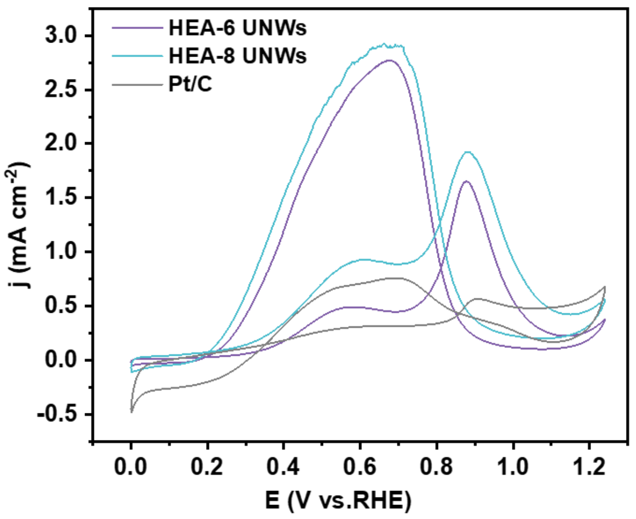

Figure S12. Cyclic voltammogram curves of HEA–8 UNWs, HEA–6 UNWs and Pt/C in 0.5 M H_2SO_4 +0.25 M HCOOH.

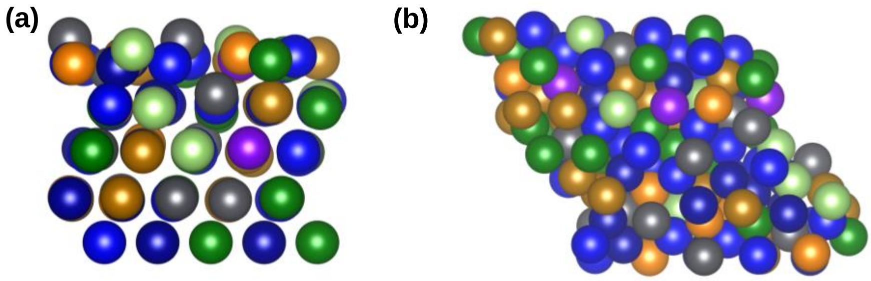

Figure S13. (a) Side view and (b) top view of HEA-8 UNWs.

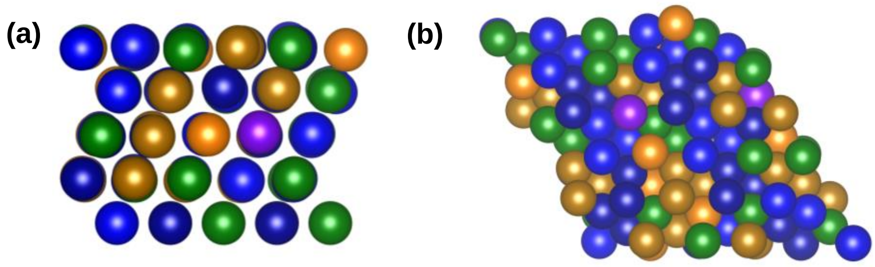

Figure S14. (a) Side view and (b) top view of HEA-6 UNWs.

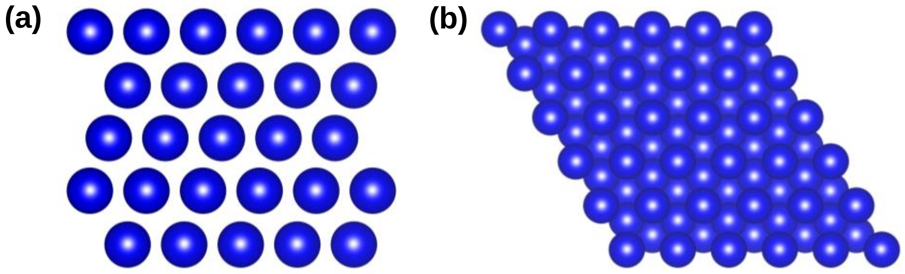

Figure S15. (a) Side view and (b) top view of pure Pt.

Table S1. Onset potential, peak current density (J_p) and various potetials at 10% J_p and 50% J_p of Pd/C, Pd NSs and Pd-PdSe HNSs

Catalyst	E_{onset} (V_{RHE})	J_p (A mg_{Pt}^{-1})	E@10% J_p (V_{RHE})	E @ 50% J_p (V_{RHE})
Pt/C	0.57	0.61	0.58	0.79
HEA-6 UNWs	0.50	1.99	0.64	0.82
HEA-8 UNWs	0.51	2.59	0.60	0.79

Table S2. The ICP-OES results before and after durability test.

HEA-8 UNWs	Pt/Ru/Ni/Co/Fe/Ga/Pb/W (ICP-OES)
before	25.5/1.5/19.9/12.5/18.0/ 5.8/10.4/6.4
after	27.3/1.2/18.8/12.1/17.1/ 6.2/12.4/4.9

Table S3 The obtained and lost number of electrons of different (The “+” represents obtaining electrons and the “-” represents losing electrons.)

Element	Pt	Ni	Fe	Co	Ru	W	Ga	Pb
Transferred electrons (e)	+0.64	-0.01	-0.32	-0.13	+0.18	-0.65	-0.39	-0.29
